# A methodology for direct quantification of over‐ranging length in helical computed tomography with real‐time dosimetry

**DOI:** 10.1120/jacmp.v12i2.3326

**Published:** 2011-01-31

**Authors:** Christopher J. Tien, James F. Winslow, David E. Hintenlang

**Affiliations:** ^1^ Department of Nuclear and Radiological Engineering University of Florida Gainesville FL USA; ^2^ Department of Radiation Physics and Imaging Physics University of Texas M.D. Anderson Cancer Center Houston TX USA

**Keywords:** helical computed tomography, over‐ranging, real‐time dosimetry, diagnostic radiology

## Abstract

In helical computed tomography (CT), reconstruction information from volumes adjacent to the clinical volume of interest (VOI) is required for proper reconstruction. Previous studies have relied upon either operator console readings or indirect extrapolation of measurements in order to determine the over‐ranging length of a scan.^(^ ^1^ ^–^ ^5^ ^)^ This paper presents a methodology for the direct quantification of over‐ranging dose contributions using real‐time dosimetry. A Siemens SOMATOM Sensation 16 multislice helical CT scanner is used with a novel real‐time “point” fiber‐optic dosimeter system with 10 ms temporal resolution to measure over‐ranging length, which is also expressed in dose‐length‐product (DLP).^(6)^ Film was used to benchmark the exact length of over‐ranging. Over‐ranging length varied from 4.38 cm at pitch of 0.5 to 6.72 cm at a pitch of 1.5, which corresponds to DLP of 131 to 202 mGy‐cm. The dose‐extrapolation method of Van der Molen et al. yielded results within 3%, while the console reading method of Tzedakis et al.^(^ ^2^ ^,^ ^4^ ^)^ yielded consistently larger over‐ranging lengths. From film measurements, it was determined that Tzedakis et al.^(^ ^2^ ^,^ ^4^ ^)^ overestimated over‐ranging lengths by one‐half of beam collimation width. Over‐ranging length measured as a function of reconstruction slice thicknesses produced two linear regions similar to previous publications.^(^ ^1^ ^–^ ^4^ ^)^ Over‐ranging is quantified with both absolute length and DLP, which contributes about 60 mGy‐cm or about 10% of DLP for a routine abdominal scan. This paper presents a direct physical measurement of over‐ranging length within 10% of previous methodologies.^(^ ^1^ ^–^ ^4^ ^)^ Current uncertainties are less than 1%, in comparison with 5% in other methodologies. Clinical implantation can be increased by using only one dosimeter if codependence with console readings is acceptable, with an uncertainty of 1.1% This methodology will be applied to different vendors, models, and postprocessing methods – which have been shown to produce over‐ranging lengths differing by 125%.^(4)^

PACS number: 87.57.qp

## I. INTRODUCTION

In helical computed tomography (CT), reconstruction information from volumes adjacent to the clinical volume of interest (VOI) must be collected in order to properly reconstruct the VOI. These additional reconstruction volumes have been variously referred to as over‐ranging lengths, overscanning volumes, or over‐ranging lengths by previous investigators, who have made indirect inferences to dose contributions from these effects.^(^ ^1^ ^–^ ^4^ ^)^ In many dosimetry evaluations of CT exams, dose contributions of the primary beam are traditionally only considered within the clinical VOI. Reconstruction algorithms will almost always require some degree of over‐ranging; thus this study aims to introduce an innovative and accurate method to directly characterize over‐ranging length with real‐time dosimetry. Typically, for a given scan length (ie, imaged volume), at least an additional one‐half of a rotation is necessary at the beginning and at the end of the scan to ensure that complete datasets are obtained for the reconstruction of the first and the last sections. Including over‐ranging lengths in the prediction of dose length products, or in Monte Carlo calculations of effective dose, will reduce underestimates of dose. ^(^ ^1^ ^–^ ^5^ ^,^ ^7^ ^–^ ^11^ ^)^ Over‐ranging lengths are especially important considerations if there are radiosensitive organs in close proximity to the clinical VOI which could have been avoided.^(^ ^7^ ^,^ ^8^ ^)^

As increasingly larger longitudinal coverage is provided by modern multisection CT scanners, over‐ranging effects increase and have to be taken into account properly. One method that addresses over‐ranging is adaptive section collimation, where parts of the X‐ray beam exposing tissue outside of the volume to be imaged are blocked in the z‐direction by dynamically adjusted collimators at the beginning and at the end of the CT scan.^(12)^ Studies by Deak et al.^(12)^ have shown dose reduction as large as 38%, but averaging 10% when dynamic collimators are used for a typical chest scan of 30 cm and pitch factor of 1.0.

Previous studies have quantified over‐ranging lengths but they rely upon either cumbersome computational studies or indirect extrapolation methods. In the studies by Tzedakis et al.,^(^ ^1^ ^–^ ^3^ ^)^ the effective dose is calculated with the Monte Carlo N‐particle (MCNP) radiation transport code. Their source uses a previously generated X‐ray spectrum emitted from line‐sources along a helical path which was benchmarked with CTDI dosimetric data, as well as z‐axis dose profiles obtained with thermoluminescence dosimeters. These Monte Carlo simulations used data displayed on the operator's console to define the simulation's scan boundaries.^(^ ^1^ ^–^ ^3^ ^)^ The proposed methodology avoids this reliance upon console measurements by determining the total length scanned using methods strictly based upon timing. Over‐ranging lengths are particularly important considerations for deterministic Monte Carlo calculations of effective dose. As noted by Tzedakis et al.,^(^ ^1^ ^–^ ^3^ ^)^ typical CT simulations assume that the exposed length is equal to the VOI length. In other words, while a simulation can accurately predict VOI dose, it will always underestimate DLP if it disregards the over‐ranging lengths, which are always a part of a clinical scan.^(13)^

An experimental physical study by van der Molen et al.^(4)^ derived the over‐ranging length with a novel extrapolation method, which is referred to as the “dose‐slope” method. In this method, the DLP was derived from measurements of a varying number of rotations. These represented the DLP due to over‐ranging and a varying size of VOI. The number of rotations which represented the VOI was then linearly extrapolated to zero rotations to represent a DLP corresponding solely to the overranging.^(4)^ This method assumes that the over‐ranging behavior stays constant at a low number of rotations. If, for example, the machine were to switch to a different reconstruction scheme, the dose‐slope method would not necessarily be accurate.

The new technique described herein has the advantage of requiring only one measurement, instead of multiple measurements necessary to create the extrapolation data. Furthermore, this technique is the first direct physical measurement of over‐ranging and, unlike the dose‐slope method, does not require extrapolations. This method is completely independent from the console readings. In addition to comparisons with the two other over‐ranging methodologies, film will be used to independently verify the exposure length.

## II. MATERIALS AND METHODS

### A. CT Scanner and real‐time dosimetry system

A Siemens SOMATOM Sensation 16 multislice helical CT scanner (Siemens AG, Forchheim, Germany) was used with a routine abdominal scan protocol. This protocol is a very diverse protocol, but was chosen to provide a direct comparison to over‐ranging measurements by Tzedakis et al. and van der Molen et al.^(^ ^1^ ^–^ ^4^ ^)^ This particular protocol permits the selection of pitches between values of 0.50 and 1.50 in increments of 0.05; rotation speeds of 0.5, 0.75, 1 and 1.5 seconds per rotation; reconstruction slice widths of 0.75, 1, 1.5, 2, 3, 4, 5, 6, 8, and 10 mm per slice; and detector collimations of 12 and 24 mm. The abdominal scan protocol was used with a tube voltage of 120 kVp and tube current of 140 mAs. The automatic exposure control was turned off for all scans.

A novel “point” tissue‐equivalent fiber‐optic coupled (FOC) real‐time dosimetry system was used for this study. While this dosimetry system accommodates up to five small dosimeter elements which can be read in real‐time with a temporal resolution of 10 ms, only three dosimeters were required for use in this study. The system performance has been previously described by Hyer et al.^(6)^ The dose linearity of the system has a correlation coefficient of 1.000 over exposures ranging from 0.16 to 57.29 mGy. Each dosimeter element is a small cylindrically‐shaped plastic scintillator 500 μm in diameter and 2 mm in length, which closely approximates a point detector. The scintillator does not suffer from angular dependencies – with the dose measured free‐in‐air at isocenter varying less than 5% over an entire revolution. In order to reduce scatter contributions for the measurements performed in this study, all dosimeters were suspended free‐in‐air.

### B. Dosimeter positioning

This methodology only involves a determination of the distance between scintillators. The maximum discrepancies between physical distances and console‐reading measurements must be no more than 1–2 mm, as stipulated by quality control guidelines.^(14)^ However, this was explicitly tested by comparing distance measured with a calibrated linear measurement device between two lead bars against a measurement from the operator's console. The difference was less than 0.50 mm. The remaining portion of the methodology is based upon the temporal spectrum obtained, where the limiting resolution of this system is 10 ms, which using a typical (pitch=1) table speed of 30 mm/s yields an uncertainty of 0.28 mm.^(6)^ Assuming even the highest table velocities (pitch=1.5), the uncertainty is slightly less than 0.50 mm. Thus assuming a high table velocity, the maximum uncertainty of the methodology is estimated as 0.7 mm – which is obtained by the sum of the console measurement precision (0.5 mm) and the timing measurement precision (0.5 mm), added in quadrature.

The unique ability of the point dosimetry system to provide real‐time information at various positions along the CT scan is exploited to analyze the temporal response of the CT equipment. Retrospective analysis of the event timeline provides all the information required in order to quantitatively evaluate the extent of over‐ranging and the corresponding dose contributions. The response is initially characterized temporally, but is readily converted into longitudinal position with information about the table feed speed, which is also verified by the experimental measurements.

**Figure 1:**
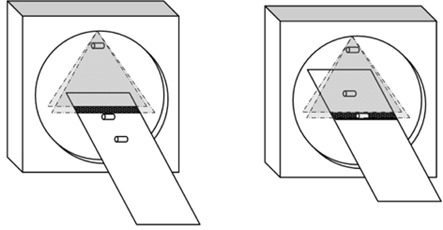
This schematic shows the dosimeter placements within the bore and upon the table. Time has transpired and the table has translated between the figures.

In the experimental setup, the first of three dosimeters is positioned in the bore at a stationary longitudinal position, while the second and third dosimeters are placed on the table, at isocenter height, and spaced 20–30 cm apart in the longitudinal direction. The bore dosimeter is suspended free‐in‐air. The only restriction is that it stays within the fan angle for the entire helical scan. In other words, the stationary bore dosimeter will always be within the scan volume and its response is used to measure the exact scan duration. The table dosimeters move through the primary beam during the scan and are only within the scan volume for a portion of the total scan. After a scout scan, the two table dosimeters are located and used to define the boundaries of the VOI for the helical scan. The temporal response of the table dosimeters is used to measure the portion of the scan exclusively in the VOI. The table speed is measured and the total scan duration and VOI scan duration are converted into distances. Over‐ranging length is determined by simply subtracting the VOI length from the total scan length. Figure 1 shows a 3D schematic of the setup – in particular the dosimeter placements within the bore and upon the table.

### C. Exposure pattern

As an absolute benchmark, the over‐ranging length was determined by measurements using film. The exact exposure pattern of the Siemens scanner was tested by scanning a computed radiography (CR) film plate at a low mA and developed at a low film speed. Each of the physical measurement techniques provides different measurements. For example, the new methodology offers a measurement of beam‐on time and small‐region table velocity measurement. (The console methodology used by Tzedakis et al. offers a beam‐on time and an overall velocity measurement.) Finally, the CR film plate offers information on the length of the scan and the pattern of the scan. The path of the X‐ray tube will be determined by its exposure pattern from the CR plate. The total exposure length will be explicitly measured and compared with the measured lengths.

### D. Clinical impact

This methodology produced over‐ranging lengths as measured in units of distance but provided no indication of clinical exposure. In order to measure dose, a number of metrics are available, such as computed tomography dose index (CTDI), dose length product (DLP) and effective dose. CTDI and its main derivatives – namely, weighted CTDI $\left({\text{CTDI}}_{w}\right)$ and volume CTDI $\left({\text{CTDI}}_{\text{vol}}\right)$ – are measured in units of milliGray (mGy); dose length product is measured in milliGray‐cm (mGy‐cm); and effective dose is measured in Sieverts (Sv).

CTDI was first introduced and defined by the U.S. Food and Drug Agency as the average dose imparted by a single axial acquisition to a standard 100 mm pencil chamber dosimeter inside a CTDI phantom over 14 CT slices.^(^ ^7^ ^,^ ^13^ ^,^ ^15^ ^–^ ^18^ ^)^ However, CTDI is expected to vary within the axial plane, so ${\text{CTDI}}_{w}$ was introduced and defined as a weighted average of the CTDI at peripheral and center locations.^(^ ^13^ ^,^ ^15^ ^)^ The advent of helical CT magnified variations along the longitudinal axis, so ${\text{CTDI}}_{\text{vol}}$ was introduced and defined as ${\text{CTDI}}_{w}$ normalized to pitch.^(^ ^7^ ^,^ ^13^ ^,^ ^15^ ^)^ ${\text{CTDI}}_{\text{vol}}$ can thus be used to express the average dose delivered to the scan volume.^(16)^ DLP was introduced to represent the total radiation exposure for a whole series of images and is defined as the product of ${\text{CTDI}}_{\text{vol}}$ and irradiated length.^(^ ^7^ ^,^ ^13^ ^,^ ^15^ ^–^ ^18^ ^)^ As explained by Dixon et al.,^(^ ^15^ ^–^ ^18^ ^)^ CTDI values are simply not accurate at the edges of the scans. Using DLP in particular would imply that the ${\text{CTDI}}_{\text{vol}}$ values would be the same for the entire length. However, for this application, the sharp dose gradients between the “on” and “off” values at the edges of the VOI allow DLP to be a useful first‐approximation to describe the over‐ranging length. The DLP reflects the total energy absorbed, and thus the potential biological effect, attributable to the complete scan acquisition. Thus, an abdomen‐only CT exam might have the same ${\text{CTDI}}_{\text{vol}}$ as an abdomen/pelvis CT exam, but the latter exam would have a greater DLP, proportional to the greater z‐extent of the scan volume.^(7)^ ${\text{CTDI}}_{\text{vol}}$ was not chosen to measure over‐ranging because it only represents a single slice.^(^ ^7^ ^,^ ^15^ ^–^ ^17^ ^)^ On the other hand, DLP is proportional to scan length and will be directly dependent on over‐ranging. In other words, over‐ranging length will provide a larger DLP with no effect upon ${\text{CTDI}}_{\text{vol}}$. Futhermore, while ${\text{CTDI}}_{\text{vol}}$ can accurately represent the dose in the central slice within a long scan length, it is not an accurate measure of dose on the edges of the scan.^(^ ^15^ ^–^ ^18^ ^)^ DLP is a useful metric specifically for over‐ranging because it is dependent upon overall length. This study thus uses DLP in addition to raw measurements of length to quantify clinical implications of over‐ranging.

## III. RESULTS

### A. Exposure pattern

The CR image is used to determine the exact length of exposure. The ROI is marked with two metallic markers on the side closest to the bore and one metallic marker on the side further from the bore. These markers are measured center‐to‐center in order to provide a length‐to‐pixel calibration factor for the image of 0.04 mm/pixel. The average length over seven images was 695 pixels (± 2.3 pixels), which converted to 27.8 cm.

If the exposed length is 27.8 cm, then the actual translation of the table would be 27.8 minus the beam collimation (half beam width at beginning and half beam width at end) or a 25.4 cm translation. In order to move 25.4 cm with the steady‐state velocity already calculated, the time required is 5.25 seconds. This agrees with the bore dosimeter timing measurement, as well as the console timing measurement.

The overall CR‐measured length of 27.8 cm is greater than the console measurement of displacement length (26.7 cm) by 1.1 cm, or about one‐half of the 2.4 cm beam collimation. Thus, as expected, exposure is occurring outside of the over‐ranging length displayed on the console. However, it is not the sum of table displacement and a full beam collimation as defined by Tzedakis et al.,^(^ ^1^ ^–^ ^3^ ^)^ but only one‐half of a beam collimation. Therefore, the over‐ranging lengths obtained by this new methodology can be compared with the Tzedakis study results adjusted by one‐half of a beam collimation width. These will be designated as “adjusted console readings”.

### B. Over‐ranging dependence upon protocol parameters

**Figure 2:**
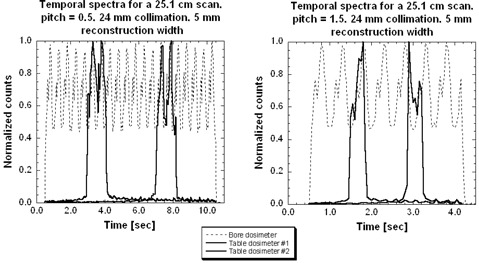
The temporal response for a 25.1 cm scan, with a low pitch of 0.5 and a high pitch of 1.5.

Figure 2 shows the response of the dosimeters as a function of time for a 25.1 cm user‐selected scan length, for the lowest pitch of 0.5 and the highest pitch of 1.5. In this figure, the width of each dosimeter's response peak represents the time within the primary beam. The time elapsed between the leading edges’ of each table dosimeters’ peak response represents the time required for the table to translate the two dosimeters into the primary beam. Table velocities are calculated by the quotient of the measured distance between table dosimeters and the time to translate between these dosimeters. There is excellent linearity between table velocity and pitch, with a correlation‐coefficient‐squared (R^2^) value of 0.999 and reproducibility for the three scans lengths performed of 19.7, 25.1, and 30 cm. This demonstrates the relationship expected between speed and pitch for a helical scan, as well as providing validation of correct dosimeter placement and measurement.

**Figure 3:**
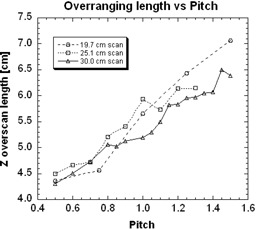
Over‐ranging length as a function of pitch shown for different scan sizes.

Figure 3 shows the over‐ranging length as a function of pitch for three different scan lengths. The over‐ranging length varies linearly with pitch, with $R^{2}$ values of 0.971, 0.942, and 0.979 for VOI scan lengths of 19.7, 25.1, and 30 cm, respectively. Over‐ranging length ranges from 4.38 cm at a pitch of 0.5 and 6.39 cm at a pitch of 1.5. Van der Molen et al.^(4)^ measured overranging lengths of 4.26 cm at a pitch of 0.5 and 6.43 cm at a pitch of 1.5. Tzedakis et al.^(2)^ measured over‐ranging lengths of 4.75 cm at a pitch of 0.5 and 7.45 cm at a pitch of 1.5. The adjusted console readings were 3.55 cm at a pitch of 0.5 and 6.25 cm at a pitch of 1.5.

**Figure 4:**
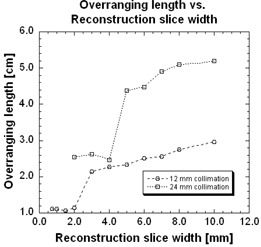
The relationship between over‐ranging length and reconstruction slice width for two beam collimations.

Over‐ranging lengths as function of reconstruction slice thicknesses for two beam collimations of 12 and 24 mm are shown in Fig. 4. Increasing the reconstruction slice width from 0.75 to 10 mm increased the over‐ranging length from 11.2 mm to 29.7 mm, respectively, using a beam collimation width of 12 mm. Increasing the reconstruction slice thickness from 2 to 10 mm using a 24 mm beam collimation width increased the over‐ranging length from 25.4 mm to 52.5 mm, respectively. Note that the smallest reconstruction slice thicknesses for collimations for 12 and 24 mm are 0.75 and 2 mm, respectively. Two distinct regions are observed in Fig. 4: a constant over‐ranging length for smaller reconstruction slice thicknesses followed by a linearly increasing over‐ranging length for larger reconstruction slice thicknesses. For example, the beam collimation of 12 mm has its constant region from 0.75 to 2 mm reconstruction slice thickness, where the over‐ranging length remains 1.11 cm ± 2.9%. Over‐ranging length is then linearly increasing with an $R^{2}$ value of 0.99 between reconstruction slice thicknesses of 3 to 10 mm. Between the two linear regions, there is an abrupt 88% increase between reconstruction slice thicknesses of 2 and 3 mm. Similarly, for beam collimation of 24 mm, over‐ranging length has its constant region from 2 to 4 mm reconstruction slice thickness, where the over‐ranging length remains 2.55 cm ± 3.3%. Over‐ranging length is then linearly increasing with a $R^{2}$ value of 0.87 between reconstruction slice thicknesses of 5 to 10 mm. Between the two linear regions, there is as an abrupt 77% increase between reconstruction slice thicknesses of 4 and 5 mm.

**Figure 5:**
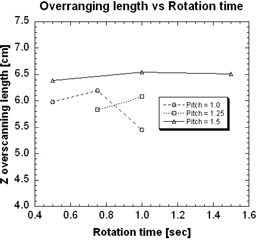
The relationship between over‐ranging length and rotation time for three different pitches.

Over‐ranging length was independent of gantry rotation speed. For these measurements, Fig. 5 shows the over‐ranging length as a function of tube rotation time from 0.5 to 1.5 seconds per rotation, for three different pitches. Over‐ranging length remained constant as a function of varying tube rotation times, even at different pitches, with measurements varying only ± 3.3%,± 2.8% and ± 1.2% for pitches of 1.0, 1.25, and 1.5, respectively.

The ROI length is set at an arbitrary length. The two table dosimeters reached 10% maximum at 1.85 and 3.85 seconds, respectively; these dosimeters were spaced 9.70 cm away. In other words, the table speed measured was 48.5 mm/s. The dosimeters were placed in the central region of the scan in order to avoid any acceleration and deceleration present at the beginning and end of the scans. This speed agrees well with the 19.7, 25.1, and 30 cm scans, which had speeds of 48.4 mm/s.

The bore dosimeter was above the 10% threshold from 0.55 to 5.80 seconds. The time of the scan is measured to be 5.25 seconds. The scanner selects a total scan length of 26.7 cm, with a predicted scan time of 5.24 seconds. This agrees well with the measured scan time.

## IV. DISCUSSION

It has been long known that in helical scanning, volumes adjacent to the clinical VOI are also exposed to the primary beam. Selecting proper beam collimation, reconstruction slice width, rotation time and pitch can help to minimize the detrimental effects of over‐ranging lengths. Clinical benefit always outweighs the potential risk in clinical diagnosis scenarios. However, the appropriate VOI selection is dependent upon the radiologist's diagnostic needs and there is certainly an amount of leeway, especially near the VOI borders. The protocol must correctly select the VOI while simultaneously ensuring correct alignment, especially if the boundaries are close to radiosensitive organs. If for example, these clinical VOI boundaries are close to radiosensitive organs or tissues such as breasts, thyroid, eye lens, or gonads, over‐ranging may result in exposure to the primary beam from its inclusion in the over‐ranging length, not to mention increased exposure to scatter. As mentioned by Tzedakis et al.,^(^ ^1^ ^–^ ^3^ ^)^ over‐ranging lengths are frequently excluded altogether from deterministic simulations which only consider the clinical VOI, thus underestimating exposure.

Over‐ranging lengths are particularly important considerations for deterministic Monte Carlo calculations of effective dose. As noted by Tzedakis et al.,^(^ ^1^ ^–^ ^3^ ^)^ typical CT simulations assume that the exposed length is equal to the VOI length. In other words, while a simulation can accurately predict VOI dose, it will always underestimate DLP if it disregards the over‐ranging lengths, which are always a part of a clinical scan.^(13)^

The results obtained by a direct measurement methodology produce numbers which agree within ± 5% of measurements obtained from console readings and within ± 3% of an indirect measurement methodology.^(^ ^1^ ^–^ ^4^ ^)^ Overall, a 5% difference in over‐ranging length is not significant in the clinical context. For example, the over‐ranging length in this helical abdominal scan was found to be around 5 cm or a maximum difference (5%) of less than 3 mm. This could be attributed primarily to the finite 10 ms timing resolution and secondarily to measurement precision of VOI boundaries. The abdominal protocol at a reconstruction slice thickness of 5 mm, 120 kVp and 140 mAs has a ${\text{CTDI}}_{\text{vol}}$ measured by the ImPACT group of 13 mGy.^(19)^ The 5% uncertainty is equivalent to a DLP of only 3.3 mGy‐cm. In terms of effective dose, a 3 mm margin would be difficult to incorporate primarily due to uncertainties from patient motion and positioning between the time of the scout scan – where the VOI boundaries are defined – and the actual helical scan. The accuracy associated with scout scan distance was tested following the QC procedure mentioned earlier, with submillimeter differences.^(14)^

It is important to consider the implications of an adjusted console reading. Specifically, if the console readings do indeed overpredict the over‐ranging length, than the DLP is similarly too large. In terms of calculations, this inaccuracy must be corrected but clinically, this can be viewed positively, in that volume exposed was smaller than originally calculated. When compared with the adjusted console measurements, the new methodology produces results which are always larger, from 2% (at pitch of 1.5) to 20% (at pitch of 0.5).

In order to reduce uncertainty, the scintillator's longitudinal axis was placed normal to the longitudinal axis in order to better approximate a point detector. Theoretically, irradiation anywhere along the 500 μm diameter would produce a signal, therefore producing an uncertainty in the VOI measurement. In the worst‐case scenario of uniform response from the scintillator, unlikely due to the cylindrical geometry, this finite diameter imparts an uncertainty of 0.5 mm, well below the limiting uncertainty imparted by the temporal resolution of 10 ms.

Analysis of the temporal response was initially conducted based on the leading edges of the detected radiation peaks. Further analysis using both leading and trailing edges on the temporal response peaks resulted in similar results within ± 2.4% of the exclusive leading‐edge technique. Subsequently, the distance between the two table dosimeters was measured and divided by the average difference in leading and trailing edges of the table dosimeter temporal response in order to give the table speed.

The table velocities were calculated from the quotient of the differences in the leading edge times obtained previously and the known distance between table dosimeters, with $R^{2}$ values of 0.999 reproducible for the three VOI scans lengths performed (19.7, 25.1, and 30 cm). These values agree to within ± 1% to the table velocity selected on the console. The table dosimeters provide a measure of average velocity over a small region, whereas the console measurement of table velocity is based on overall scan length and overall scan time. The table velocity measured over a small region should agree well with the table velocity measured over the entire scan by the console, which implies that the acceleration and deceleration regions are negligible. Figure 3 shows the linear relationship between over‐ranging length and pitch for different scan sizes, with $R^{2}$ values of 0.971, 0.942, and 0.979 for VOI scan lengths of 19.7, 25.1, and 30 cm, respectively. Our methodology thus demonstrates the expected independence of over‐ranging length upon different individual clinical VOI lengths; this will have obvious effects upon normalizing the (constant) over‐ranging lengths to the (varying) clinical VOI lengths. The DLP measured for the three scan lengths of 19.7, 25.1, and 30 cm were 256, 326, 390 mGy‐cm. Figure 3 shows over‐ranging lengths ranging from 4.38 to 6.39 cm, with an average around 5.5 cm; these correspond to DLP of 131 and 192 mGy‐cm, and an average of 165 mGy‐cm. The results are summarized in Table 1.

**Table 1:** Comparison of over‐ranging length and pitch for differing measurement methodologies.

*Pitch*	*New Method's Average Value [mm]*	*Dose‐slope (van der Molen et al.) [mm]*	*Console Reading (Tzedakis et al.) [mm]*
.5	43.9	42.6	47.5
.75	45.6		53.0
1	54.8	53.5	63.0
1.25	62.0		70.5
1.5	67.3	64.3	74.5

One advantage of this new methodology is its complete independence from console measurements. Specifically, the methodology prescribes a measurement of table velocity for each and every scan instead of relying upon the velocity and time calculated by the console. The Siemens scanner itself predicts the time required for the scan and displays the “scan time” before the scan is actually done. This is presumably done with a speed look‐up table which depends on a combination of tube rotation time, pitch, detector collimation and reconstruction slice thickness.

It is possible to refine the methodology to operate with only the bore dosimeter and eliminate the table dosimeters if independent speed measurements are not desired. If the system is operated in one‐dosimeter mode, the degree of precision and accuracy is exceptional and is limited only in the time resolution of one detector. With our current design using 10 ms resolution, the uncertainty is less than one‐half of a millimeter (for pitch=1 the table velocity is ~ 48 mm/s). The console‐reading measurements and physically‐measured distances must differ by no more than 1–2 mm as stipulated by QC guidelines; therefore, the maximum error would be seen in a smaller scan, and is 1.1% for a 19.1 cm scan. In fact, this impressive number could be further improved by faster PMT binning. Using the one‐dosimeter mode would make it much simpler to use clinically and has exceptional uncertainty; but, as discussed, this will eliminate the complete independence of the dosimetry system.

Figure 4 shows two distinct linear regions for smaller and larger sections – this reconstruction slice width dependence has been measured by both van der Molen et al. and Tzedakis et al.^(^ ^1^ ^–^ ^4^ ^)^ These two regions arise from different reconstruction schemes used by Siemens scanners for smaller (cone‐beam‐corrected mode) and larger sections (z‐filtering).^(4)^ Specifically, the “cone‐beam‐corrected mode” regime is used for smaller thicknesses (0.75–2 mm reconstruction slice thickness for 12 mm beam collimation; 2 to 4 mm for 24 mm), while the faster Siemens “z‐filtering algorithm” is used for larger reconstruction slice thicknesses (3–10 mm for 12 mm; 5–10 mm for 24 mm).

This methodology demonstrates the linear dependence of over‐ranging length on pitch which has been observed in previous publications.^(^ ^1^ ^–^ ^4^ ^)^ With a routine abdominal protocol, over‐ranging length is clinically relevant and can be expected to contribute an average of 10% extra dose, or a DLP around 60 mGy‐cm (30 cm scan length with a ${\text{CTDI}}_{\text{vol}}$ of 20 mGy). Overall, the overranging lengths for varying pitch, reconstruction slice width and beam collimation measured using this methodology agree within 5% with measurements by van der Molen et al.^(4)^ Further agreement in over‐ranging lengths was observed with console‐reading results obtained by Tzedakis et al.^(^ ^1^ ^–^ ^3^ ^)^ for 12 mm beam collimations; however, the over‐ranging lengths obtained with 24 mm beam collimations were slightly lower than the console‐reading results obtained by Tzedakis et al. by around 5%–10%.^(^ ^1^ ^–^ ^3^ ^)^ Regardless, assuming a routine abdominal protocol, a 5% discrepancy in over‐ranging length is equivalent to a DLP of only 3.3 mGy‐cm. The overestimate of effective dose by Monte Carlo methods is mentioned by van der Molen et al.^(4)^ and attributed to the simulated beam profile, which does not produce any noticeable differences until a larger beam collimation of 24 mm is used. (This is discussed in more detail by van der Molen et al.^(4)^) However, the CR exposure pattern test perform suggests that the Tzedakis studies overestimated the over‐ranging length simply because a whole beam collimation width was added rather than one‐half of a beam collimation width.

Gantry rotation speed was not expected to contribute to over‐ranging length because the actual scan length required is determined by the amount of reconstruction information required. If the reconstruction scheme remains constant, this should remain fixed. In other words, it should not change depending upon the speed of the gantry, unless the reconstruction scheme changed as a function of gantry rotation speed. This parameter was included in order to measure over‐ranging for any operator‐controllable parameters.

This methodology only involves one physical measurement: distance between scintillators. According to QC guidelines, any discrepancies between measured and physical distances should be no more than 1–2 mm.^(14)^ This was explicitly tested by comparing distance measured with a calibrated linear measurement device between two lead bars against a measurement from the operator's console. The difference was less than 0.50 mm. The remaining portion of the methodology is based upon the temporal spectrum obtained, where the limiting resolution of this system is 10 ms which, using a typical (pitch=1) table speed of 30 mm/s yields an uncertainty of 0.28 mm.^(6)^ Assuming even the highest table velocities (pitch=1.5), the uncertainty is slightly less than 0.50 mm. Thus assuming a high table velocity, the maximum uncertainty of the methodology is estimated as 0.7 mm – which is obtained by the sum of the console measurement precision (0.5 mm) and the timing measurement precision (0.5 mm), added in quadrature.

There is good general agreement with other published techniques. However, this method produces better precision than van der Molen et al. and also addresses a significant error made by Tzedakis et al. The technique used by Van der Molen et al. involved an extrapolation method with a linear‐fit confidence interval of 0.95. The precision of the data used in order to perform this extrapolation was not discussed. Therefore, it is assumed that the error bars on those data were considered negligible by van der Molen et al., giving a precision on the order of 5%, compared with maximum uncertainty of this new methodology of 1.1%.

## V. CONCLUSIONS

In this methodology, using two table dosimeters provided a measurement of average velocity over a small region, whereas the console determines table velocity based on overall scan length and overall scan time. The table velocity measured over a small region agrees well with the overall measured table velocity, which is important because it demonstrates that the acceleration and deceleration regions are negligible. If implemented clinically, this method could potentially be adapted to operate in conjunction with the console measurements of speed, thus eliminating the need for the two dosimeters. This would also reduce the number of measurements required, but introduce a strong codependence upon console accuracy.

This method is limited in that it may not work for modern scanners which utilize dynamic collimators. However, neither the console readings by Tzedakis et al. nor the extrapolation method by van der Molen et al. would work in this situation. A new method would need to be developed in order to account for the partially open or closed collimators.

Over‐ranging length can result in quite significant contributions to patient exposure. For a routine abdominal protocol, over‐ranging length was determined to be clinically relevant with an average contribution of 10% extra dose, or a DLP around 165 mGy‐cm. In spite of this, DLP is traditionally measured only for the VOI, omitting over‐ranging length. This technique has an advantage over console‐reading simulations because it is scanner‐independent and does not require benchmarking or confidential proprietary information notably bowtie filter spectra. Furthermore, this method requires a single measurement, while current physical methods require multiple measurements to establish an extrapolation baseline. Lastly, this method avoids assumptions regarding immediate irradiation during table translation. This quick and direct methodology can be easily implemented in order to include over‐ranging lengths for proper calculations of total DLP. Over‐ranging length results have been measured for a Siemens SOMATOM Sensation 16. Future research will include different vendors, scanner models and post‐processing methods – which have been shown to change over‐ranging values by as much as 125%.^(4)^
